# Bowel and Small Joint Involvement in Granulomatosis With Polyangiitis: A Case Report

**DOI:** 10.7759/cureus.68997

**Published:** 2024-09-09

**Authors:** Muhammad Bilal Mohsin, Uswah Rasool, Laiba A Rana, Tahir M Hashmi

**Affiliations:** 1 Internal Medicine, Shifa International Hospital Islamabad, Islamabad, PAK; 2 Rheumatology, Shifa International Hospital Islamabad, Islamabad, PAK

**Keywords:** anca associated vasculitis, bowel involvement, c-anca/proteinase 3-positive granulomatosis with polyangiitis, granulomatosis with polyangiitis (gpa), negative rheumatoid factor polyarthritis, small joint arthritis

## Abstract

We discuss the case of a 45-year-old male with complaints of abdominal pain, loose stools with on and off melena, and multiple joint pains bilaterally. The patient reported having similar episodes in the past, with slight variations in the symptoms and relief with short courses of steroids. Initially, a workup was done to identify an infectious etiology of the diarrhea. However, no such cause was identified. Similarly, an autoimmune profile was ordered to investigate the patient’s joint complaints, albeit with no conclusive findings. His renal function tests and urinalysis showed findings indicative of an acute kidney injury. This prompted an antineutrophil cytoplasmic antibody (ANCA) profile, which was positive for c-ANCA. A diagnosis of granulomatosis with polyangiitis (GPA) was made, and the patient was started on pulse steroid and immunomodulator therapy with improvement in the patient’s condition. This case is atypical due to its involvement of the gastrointestinal system, which is relatively rare, as well as polyarthritis of the small joints.

## Introduction

Typically, granulomatosis with polyangiitis (GPA) is known to have characteristic involvement of the kidneys and upper and lower airways [1]. Other systemic involvement has also been previously noted in past studies, making diagnosis challenging when such atypical presentations arise [2]. Our patient is seen to have gastrointestinal involvement alongside skin, kidneys, and joints. Gastrointestinal involvement in association with GPA is unusual but not unheard of, having been previously reported in the literature [1,3-5]. Joint involvement has also been seen in association with GPA. However, this typically involves large joints like the knees and ankles [2]. Our patient had a peculiar symmetric involvement of the small joints of his hands, similar to rheumatoid arthritis. We aim to report these unusual findings.

## Case presentation

**Figure 1:**
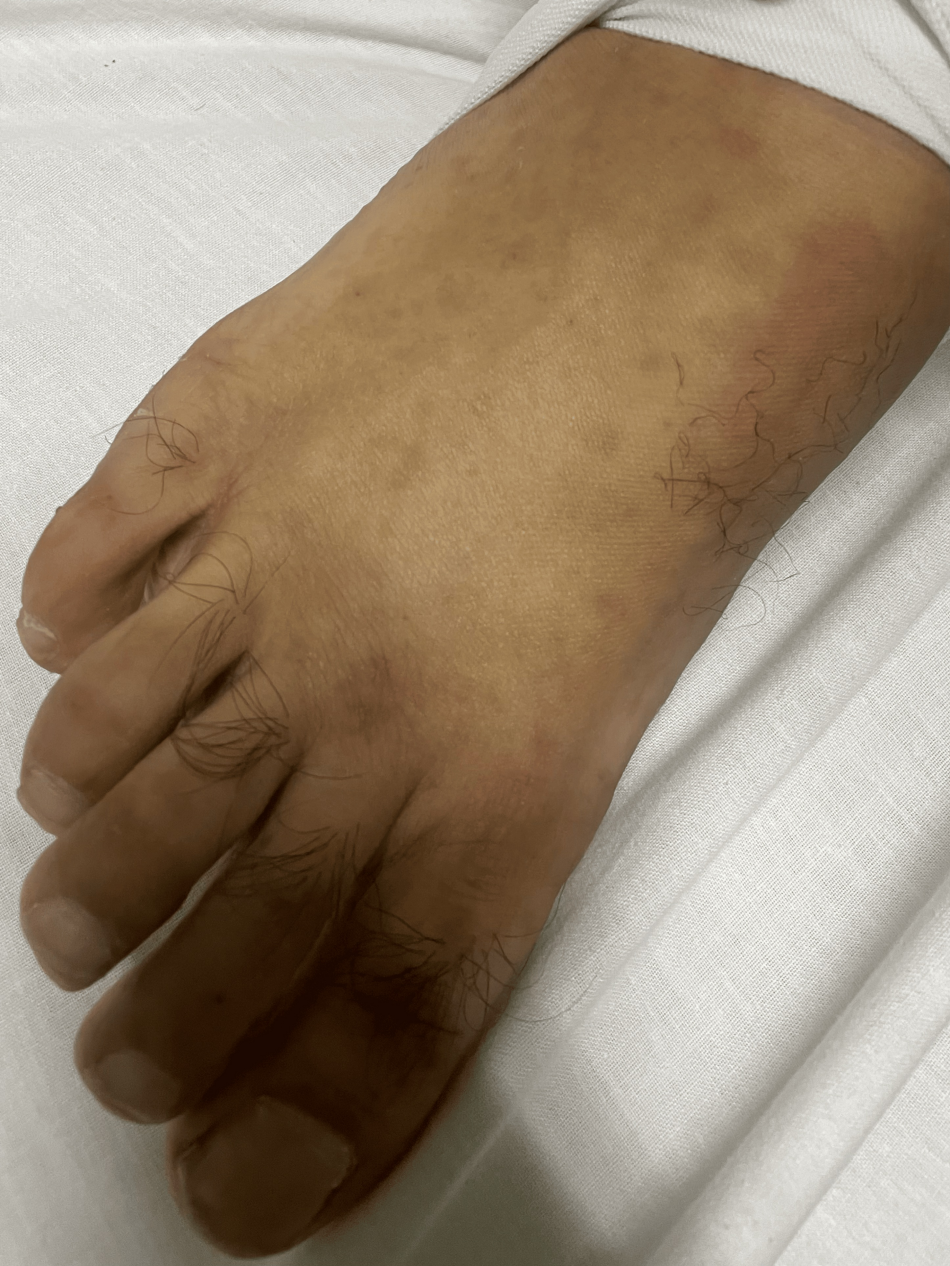
Faded diffuse purpuric rash on the right foot of the patient.

A 45-year-old male presented to the emergency department with complaints of paraumbilical pain and multiple episodes of loose stools with on and off melena for the past day. He also complained of multiple joint pains, mainly involving the interphalangeal joints and the right elbow. Examination revealed paraumbilical tenderness along with tenderness and inflammation in the proximal interphalangeal joints bilaterally. Additionally, pedal edema was noted, and a diffuse purpuric rash (Figure 1) was seen on the lower limbs, buttocks, and upper limbs.

He has had similar episodes of polyarthralgia with an accompanying rash in the past. The first of these was four months ago. At the time, he was managed conservatively with steroids, and the skin rash resolved spontaneously. His management was complicated due to bilateral otitis media, which had to be treated with antibiotics. The second of these episodes was one month ago, this time with hematochezia. During his hospitalization, he had to undergo three sessions of hemodialysis due to uremia secondary to an acute kidney injury. His urinalysis was remarkable for the presence of blood and protein, and numerous red blood cells were visualized. Additionally, his hemoglobin was found to be 5.9 g/dl, and he was transfused five units of packed red blood cells. An esophagogastroduodenoscopy revealed mild fungal esophagitis and mild pan-erosive gastritis, and a colonoscopy showed black stools with clotted blood. Additionally, a computed tomography scan of the abdomen indicated mild thickening in segments of the descending and the sigmoid colon with skip lesions. Due to the fungal esophagitis, a workup for HIV was done; however, this came back negative. He was managed along the lines of Henoch-Schönlein purpura with steroids and antibiotics.

During his current hospitalization, his initial lab findings are given in Table 1. 

**Table 1:** Patient labs at the time of admission Na, sodium; K, potassium; HCO_3_, bicarbonate; BUN, blood urea nitrogen; Cr, creatinine; eGFR, estimated glomerular filtration rate; WBC, white blood cells; Hb, hemoglobin; MCV, mean corpuscular volume; ESR, erythrocyte sedimentation rate; CRP, C-reactive protein

Labs	Value	Normal Range	Unit
Na	133	135-145	mEq/L
K	3.6	3.5-5.2	mEq/L
HCO_3_	21	22-28	mEq/L
BUN	17	6-24	mg/dL
Cr	1.5	0.7-1.3	mg/dL
eGFR	52	>90	mL/min/1.73 m2
Albumin	3.0	3.5-5.5	g/dL
WBC	10.6	4.5-11.0	x10^9 ^/L
Hb	9.5	13.0-17.0	g/dL
MCV	82.0	80-90	fL
Platelets	403	150-400	x10^9 ^/L
ESR	140	<20	mm/hour
CRP	99.06	<0.3	mg/dL

Stool routine examination (R/E) was unremarkable, with no visualization of RBCs (Table 2).

**Table 2:** Stool routine examination of the patient at the time of admission RBC, red blood cell; WBC, white blood cell

Parameter	Result	Reference
Color	Brown	Brown
Consistency	Loose	Solid
Gross Blood	Negative	Negative
Mucus	Negative	Negative
Ova	Not seen	None
Cyst	Not seen	None
Worm	Not seen	None
RBCs	Nil	Nil
WBCs	Nil	Nil

The patient's workup for Clostridium difficile (Table 3) and his abnormal urine routine examination findings are shown in Table 4. 

**Table 3:** Results from Clostridium difficile antigen and toxin testing GDH, glutamate dehydrogenase

Parameter	Result	Reference
GDH antigen	Not detected	Absent
Toxin (A)	Not detected	Absent
Toxin (B)	Not detected	Absent

**Table 4:** Abnormal urinalysis findings The presence of 2+ blood indicates significant microscopic hematuria supplemented by the visualization of 20-25 red blood cells per high-power field. The 2+ protein indicates proteinuria corresponding to approximately 100 mg of protein per dL of urine. The casts visualized were described as being granular and hyaline in nature. HPF, high power field

Parameter	Result	Reference
Appearance	Turbid	Clear
Protein	2+	Negative
Blood	2+	Negative
Red Blood Cells	20-25	0-2/HPF
White Blood Cells	12-15	1-2/HPF
Cast	Present	NIL

His elevated creatinine and decreased GFR, along with his urinalysis report, indicated an acute kidney injury. He underwent a renal ultrasound, which showed no abnormalities. His raised ESR and CRP, alongside polyarthritis and a purpuric rash, prompted investigations for an autoimmune cause. The results of this workup are given in Table 5.

**Table 5:** Patient's immunological workup findings ANA, antinuclear antigen; dsDNA antibodies, double-stranded deoxyribonucleic acid antibodies; RF, rheumatoid factor; Anti-CCP, anti-cyclic citrullinated peptide; c-ANCA, cytoplasmic antineutrophil cytoplasmic autoantibody; p-ANCA, perinuclear antineutrophil cytoplasmic autoantibody; IgE, immunoglobulin E

Labs	Value	Normal range	Unit
ANA	Negative	Negative	-
dsDNA antibodies	<0.1	<5	AU
RF quantitative	197	<14	IU/ml
Anti-CCP	8.8	<17	U/ml
c-ANCA	100	<5	AU
p-ANCA	0.1	<5	AU
C3 complement	1.49	0.90-1.80	g/L
C4 complement	0.18	0.10-0.40	g/L
IgE	338.0	In adults <100	IU/ml
Fibrinogen	552.10	199-463	mg/dl

**Figure 2:**
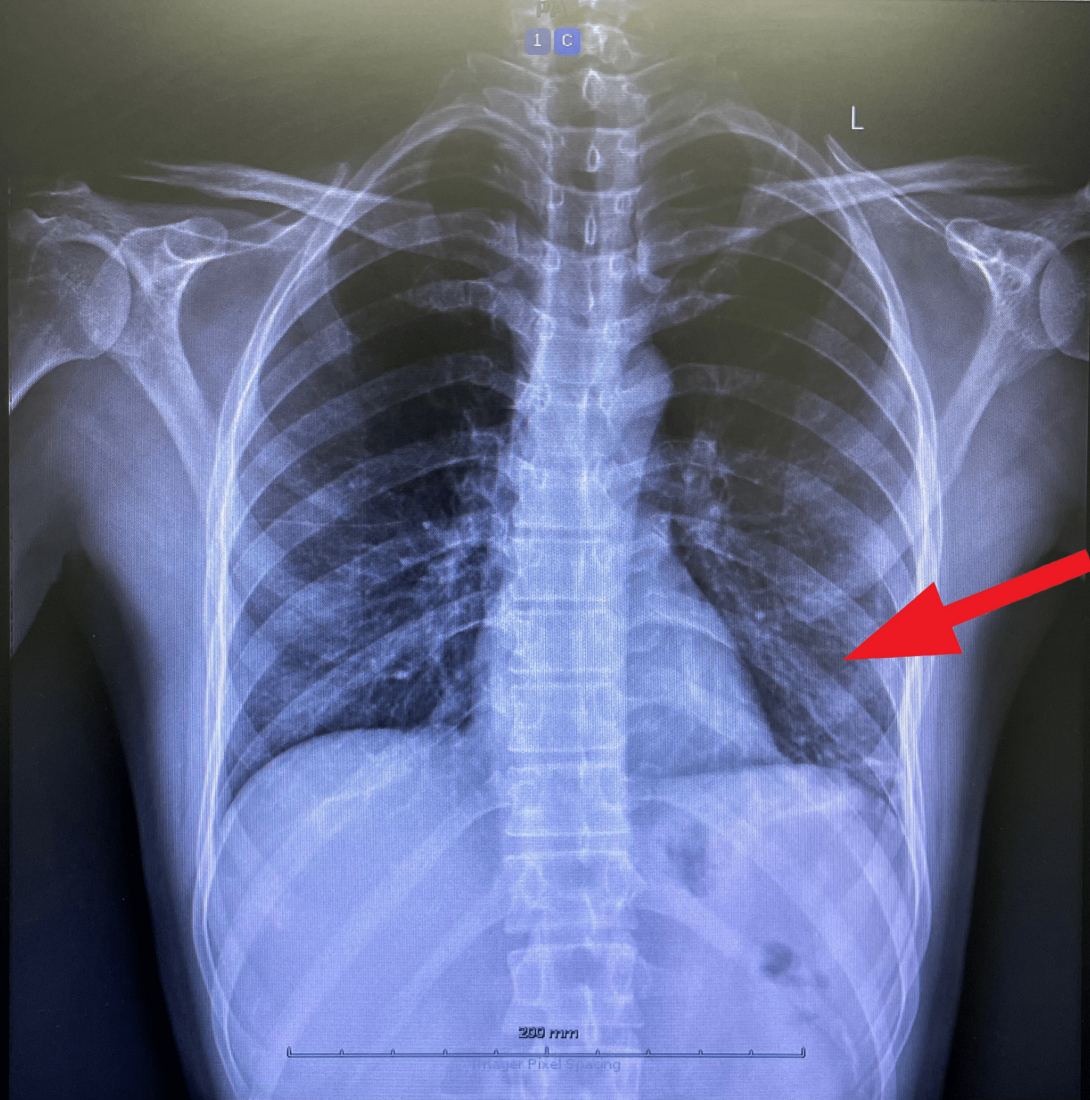
Patient's chest X-ray Atelectatic changes were noted in the left lung base (red arrow).

The elevated c-ANCA pointed to a diagnosis of granulomatosis with polyangiitis (GPA), and a chest X-ray showed a few atelectatic changes to the left lung base (Figure 2).

He was started on Solu-Medrol 500 mg intravenously for four days, followed by two doses of cyclophosphamide 650 mg (10 mg/kg) two weeks apart. This led to an improvement in the patient’s condition. He was discharged on oral prednisolone 60 mg and was seen for follow-up in the outpatient clinic. The oral prednisolone was tapered down to 50 mg, and he was started on azathioprine 50 mg with a good response.

## Discussion

GPA is a small-to-medium vessel disease characterized by necrotizing granulomatous inflammation [1]. Most often, it is characterized by the involvement of the upper and lower respiratory tracts alongside the kidneys [1]. However, it is not uncommon to see a wide variety of other systemic manifestations of the disease in these patients, and the heterogeneity of symptoms makes the clinical diagnosis of GPA challenging [2]. 

Although the gastrointestinal system has been reported to be involved in various small and medium vessel vasculitides, its involvement in GPA is still rare, involving anywhere from 5-24% of cases with GPA, most often seen in males [1,3-5]. A study by Pagnoux et al. evaluating gastrointestinal involvement in patients with small and medium vessel vasculitides found that the most commonly seen GI symptoms include abdominal pain (97%), nausea or vomiting (34%), diarrhea (27%), melena or hematochezia (16%), and hematemesis (6%) [6]. The abdominal pain and diarrhea, alongside the on-and-off episodes of melena and hematochezia experienced by our patient, were in line with these findings. Initially, it was thought that these symptoms were due to Crohn’s disease, which can be challenging to differentiate from GPA given its similar involvement of the gastrointestinal tract, skin, and joints [1]. Eventually, the delineating factor was the presence of c-ANCA antibodies, which are characteristically associated with GPA and were found to be positive in our patient, favoring the diagnosis.

In addition, our patient experienced symmetric polyarthritis involving the small joints of the hands as well as his right elbow at the time of presentation. It has been noted that arthritis associated with GPA typically affects large joints, particularly the knee and ankle joints. To our knowledge, this atypical involvement of small joints has been previously reported in six patients with GPA who had a negative anti-CCP [2]. Thereby, it was noted that early arthritis in male patients with a negative anti-CCP can be indicative of GPA. Patients with such unusual presentations often get misdiagnosed initially, as seen in our patient as well. The delay in appropriate treatment increases morbidity and disease progression. Although an association has not been made between the patient's gender and having this atypical presentation, it is to be noted that similar to all six of the patients discussed in the literature previously, our patient is also male [2]. Therefore, we highlight the importance of considering GPA, particularly in male patients who develop symmetric arthritis of the small joints and have negative anti-CCP.

## Conclusions

We have described a case of GPA with atypical symmetric involvement of the small joints. Although polyarthritis has been previously reported in association with GPA, it is commonly noted to involve large joints. Our patient presented with abdominal pain, loose stools with on and off melena, and multiple joint pains bilaterally. He had similar episodes in the past, which were treated with steroids. Workup revealed elevated c-ANCA, leading to the diagnosis of GPA. Pulse steroid therapy and cyclophosphamide were initiated, and the patient showed improvement. With this case, we aim to highlight arthritis of the small joints in male patients with a negative anti-CCP as one of the sequelae of GPA. Therefore, it is important to consider this as a differential to ensure accurate diagnosis.
